# Editorial: Exploring untapped potential: innovations in drug repurposing

**DOI:** 10.3389/fphar.2026.1996284

**Published:** 2026-09-29

**Authors:** Chong Xu, Qian Zhou, Roberto Paganelli

**Affiliations:** 1 School of Basic Medical Science and Clinical Pharmacy, China Pharmaceutical University, Nanjing, China; 2 College of Food Science and Light Industry, Nanjing Tech University, Nanjing, China; 3 Saint Camillus International Medical University- Internal Medicine, Rome, Italy; 4 Cromaton Laboratory, Allergy & Immunology, Vignola, Modena, Italy

**Keywords:** computational drug discovery, drug repurposing, drug safety, mechanism of action, therapeutic innovation, translational pharmacology

Drug repurposing occupies an unusual position in drug development. It begins with an advantage that conventional discovery does not have: prior knowledge. For an approved or otherwise well-characterized compound, information may already exist on formulation, pharmacokinetics, tolerability, manufacturing, and human exposure. This can shorten parts of the development pathway, but it does not remove the central questions that determine whether a therapy is useful. A drug effective in one setting is not necessarily effective, appropriately dosed, or sufficiently safe in another. Repurposing therefore succeeds only when prior pharmacological knowledge can be connected to a credible disease mechanism and then tested under conditions relevant to the proposed indication.

The contributions to this Research Topic reflect how broad that process has become. Some examine established drugs in new diseases; others identify candidate indications computationally or through screening, define mechanisms that may support future repositioning, explore natural products and traditional medicines from a repurposing perspective, or address clinical effectiveness and toxicity. Not every study represents drug repurposing in its narrowest regulatory definition. Taken as a Research Topic, however, the articles map the scientific terrain within which credible repurposing decisions are made: candidate identification, mechanistic plausibility, disease context, experimental validation, safety, and clinical translation.

At the discovery end of this spectrum, computational methods are increasingly being used to identify relationships that are difficult to recognize from individual datasets. KGRDR integrates biomedical knowledge-graph information with graph-regularized similarity information to predict drug-disease associations, with Alzheimer’s and Parkinson’s diseases used as case studies (Luo et al.). A complementary experimental strategy was applied in an FDA-approved drug library screen using an ACE2-dependent SARS-CoV-2 pseudovirus-entry assay, which identified 18 active drugs or candidates, including four compounds not previously reported in that assay (Singh et al.). The distinction between these approaches is useful. Computational models can reduce a large candidate space efficiently, whereas phenotypic screening tests functional activity without requiring complete prior knowledge of the mechanism. Neither approach, by itself, establishes a new therapy; both are most valuable when they yield candidates that can be tested mechanistically and in disease-relevant systems.

Mechanistic work provides the bridge between an unexpected drug-disease association and a defensible repurposing hypothesis. A study of hnRNP A2B1 combined radiation-exposed endothelial-cell experiments with molecular docking and molecular-dynamics analyses to examine this RNA-binding protein as a candidate target for radiomodulatory drugs (Lubinets et al.). The review of metabolic abnormalities in vitiligo approached the same problem from the opposite direction, considering how oxidative stress, mitochondrial dysfunction, and altered lipid and glucose metabolism might expose opportunities for existing metabolic interventions (Paganelli et al.). Repurposing need not therefore begin with a drug in search of a disease. It can begin with a disease mechanism and ask which established pharmacologies intersect with it.

Several contributions provide more direct examples of established drugs being evaluated outside their conventional therapeutic roles. Esomeprazole inhibited proliferation of patient-derived scleroderma fibroblasts and induced G1 arrest, accompanied by increased p21 and reduced CDK1/CDK2 signaling (Khan et al.). Clopidogrel attenuated high-fat diet-induced hepatic steatosis in mice, with enhanced AMPK/ACC phosphorylation and broader changes in lipogenic, inflammatory, and fibrotic pathways (Tai et al.). Oseltamivir phosphate was examined in hepatocellular carcinoma, where its antitumor effects were linked to NEU1 inhibition and PLIN2-associated lipophagy (Chen et al.). In experimental autoimmune myocarditis, sivelestat reduced inflammatory injury and neutrophil extracellular-trap formation, while integrated pharmacological analyses implicated PI3K-Akt-associated signaling among the affected mechanisms (Ren et al.). These studies matter not simply because known compounds produced additional biological effects, but because each attempted to explain why those effects might be relevant to the new disease context.

Oncology remains particularly receptive to this logic because resistance, pathway redundancy, and combination treatment create multiple points at which established agents may acquire new value. A review of dimethyl fumarate assessed evidence supporting its use as an anticancer agent, chemosensitizer, and potential modifier of treatment-related toxicity (Zhang et al.). Experimental work on salidroside showed that it enhanced 5-fluorouracil activity against hepatocellular carcinoma and implicated YIPF5-associated mitophagy in this interaction (Sun et al.). These represent two distinct forms of therapeutic redirection: assigning a new disease context to an established drug and using a known bioactive compound to alter response to a standard therapy.

The Opinion article on natural depsipeptides isolated through iChip technology extends the discussion into a more exploratory space. It considers whether compounds such as teixobactin and clovibactin, developed primarily in an antibacterial context, might be investigated for breast cancer and proposes that computational approaches could assist candidate prioritization (Fhatonah et al.). Here the distinction between hypothesis and evidence is essential. The proposed anticancer application remains a direction for experimental investigation rather than an established therapeutic effect. Repurposing research benefits from such hypotheses, but their evidentiary status should remain explicit until target engagement, relevant exposure, efficacy, and safety are demonstrated.

Therapeutic response also depends on the biological environment in which a drug is used. The study examining obesity and immune-checkpoint therapy in non-small-cell lung cancer is not a conventional repositioning study, yet it is highly pertinent to this Research Topic. The authors linked obesity-associated enhancement of immunotherapy response to altered glycolytic metabolism, lactate handling, histone lactylation, and PD-1 regulation in CD8^+^ T cells (Wang et al.). Such findings draw attention to a point that can be overlooked when candidates are selected primarily from drug-target relationships: metabolic state, comorbidity, immune phenotype, and other patient-level variables may substantially modify response. A repurposed therapy may therefore be most credible in a biologically defined subgroup rather than across an entire diagnostic category.

A similarly broad view emerges from the natural-product and traditional-medicine studies in the Research Topic. Total phenols from Hemerocallis citrina leaves improved depressive-like phenotypes in chronic unpredictable mild stress-exposed rats and were associated with changes across the microbiota-gut-brain axis (Wang et al.). An ethanol extract of Portulaca oleracea attenuated experimental atherosclerosis while modulating cholesterol uptake and efflux pathways (Chen et al.). Gambogic acid ameliorated DSS-induced colitis and reduced intestinal epithelial necroptosis in association with HSP90 inhibition and decreased RIPK1/RIPK3/MLKL phosphorylation (Wang et al.). Honey-fried licorice decoction reduced atrial-fibrillation susceptibility in an experimental model and was linked to suppression of the NOX2-ROS-TGF-β1 pathway (Qin et al.). These studies broaden the chemical and therapeutic space from which repositioning hypotheses may arise, but they also sharpen an important methodological problem: chemically complex preparations require reproducible characterization, exposure information, and increasingly precise attribution of active constituents and targets.

The review on drug repurposing in traditional Chinese medicine places this challenge in a wider framework, arguing for a progression from empirical therapeutic observations toward systems-level analysis, preclinical validation, and clinically testable strategies (Wu et al.). Traditional use can generate a useful therapeutic hypothesis, but historical familiarity cannot substitute for a traceable pharmacological evidence chain. The same standard should apply across conventional small molecules, natural products, and multicomponent preparations: composition and exposure must be defined well enough for mechanism, efficacy, and safety to be interpreted reproducibly.

Neurological studies in the Research Topic further illustrate how repositioning opportunities can emerge from disease mechanisms and comorbid conditions. Dietary dihydroquercetin ameliorated age-related cognitive impairment in mice in association with reduced neuroinflammation, apoptosis, and TLR4/NF-κB/NLRP3/CASP1-related pyroptotic signaling (Zeng et al.). In 5×FAD mice, pentylenetetrazole kindling accelerated Alzheimer-like neuropathology, whereas long-term carbamazepine treatment attenuated seizure-associated worsening of pathological changes and ERK-DAPK activation (Zou et al.). The latter study is especially instructive because it asks whether treating one established neurological disorder might also modify processes that contribute to a coexisting neurodegenerative disease. The result provides a preclinical rationale for further study, but it does not establish disease modification in patients with Alzheimer’s disease.

Safety must be considered with the same discipline as efficacy. The study of brigatinib in human immortalized keratinocytes did not propose a new indication; instead, it characterized inhibition of proliferation and induction of apoptosis, with involvement of growth-factor signaling and the PI3K/AKT pathway (Yang et al.). Its relevance to repurposing is direct. Existing clinical experience can reduce uncertainty about a drug, but safety cannot simply be transferred from one indication to another. Changes in dose, duration, combination therapy, tissue exposure, comorbidity, and patient susceptibility may all alter risk. Mechanistic toxicology is therefore part of deciding whether a proposed new use is pharmacologically realistic, not an ancillary exercise performed after efficacy has been established.

Clinical evidence introduces a different level of uncertainty. In a retrospective MIMIC-IV analysis, dexmedetomidine exposure in patients with sepsis-induced myocardial injury was associated with lower short- and longer-term mortality (Liu et al.). The association is clinically relevant but cannot establish causality, and prospective confirmation remains necessary. A randomized, double-blind, placebo-controlled protocol evaluating 5% lidocaine-medicated plaster after knee replacement represents a later translational step: the study is designed to determine whether an established local anesthetic intervention can prevent chronic postsurgical pain, but the protocol itself does not demonstrate benefit (Yuan et al.). At another level of evidence, a network meta-analysis compared pharmacological treatments for idiopathic pulmonary fibrosis on clinically relevant lung-function outcomes (Yin et al.). Comparative evidence synthesis is distinct from candidate discovery, but it addresses the question that ultimately determines the value of a reused therapy: how does it perform relative to available alternatives?

Across these contributions, a consistent lesson emerges. Drug repurposing should not be regarded as a way to bypass drug development. It is better understood as a way to begin from a different point. Existing knowledge may reduce uncertainty about chemistry, formulation, pharmacology, or human exposure, but the new indication still requires its own evidence. Computational prediction is not target validation; target engagement is not therapeutic efficacy; activity in an animal model is not clinical benefit; and prior approval is not proof of safety under a different therapeutic context.

The field is therefore likely to advance less by increasing the number of proposed drug-disease pairs than by improving the evidence connecting them. Knowledge graphs and machine learning can make candidate selection more efficient, but predictions should lead to experiments capable of falsifying the proposed mechanism. Systems pharmacology and multi-omics can help resolve complex responses, but mechanistic breadth should not substitute for causal validation. Pharmacokinetic exposure and target engagement need to accompany efficacy studies more routinely, particularly when experimentally effective concentrations may not be clinically achievable. Safety should also be reconsidered early when the intended population, treatment duration, or combination regimen changes.

The studies collected in this Research Topic span much of this continuum, from candidate generation and mechanistic exploration to preclinical validation, toxicology, evidence synthesis, and clinical investigation. Their heterogeneity is informative. It shows that repurposing is no longer a single experimental strategy, but a framework for asking whether existing pharmacological knowledge can be redirected toward unmet therapeutic needs. The most persuasive opportunities will not be those supported merely by the familiarity of a compound. They will be those in which mechanism, exposure, efficacy, patient context, and safety converge strongly enough to justify translation.

